# Fatal Consequences of Head Trauma: A Case Report of Venous Air Embolism Complicated by Substance Abuse and Liver Disease

**DOI:** 10.7759/cureus.71754

**Published:** 2024-10-18

**Authors:** Antun Ferencic, Martin Mervic, Tomaz Zupanc

**Affiliations:** 1 Institute of Forensic Medicine, School of Medicine, University of Ljubljana, Ljubljana, SVN

**Keywords:** head injury, liver disease, substance abuse, traumatic injuries, venous air embolism

## Abstract

Venous air embolism is a rare but potentially fatal complication arising from traumatic injuries or medical procedures, characterized by the intravascular introduction of air leading to the formation of gas emboli within the pulmonary vasculature and cardiac chambers. Here, we present the case of a 34-year-old female with a history of substance abuse who sustained a head injury during an altercation. Despite initial resistance to medical assistance due to intoxication, she eventually received treatment for a significant head wound. A postmortem examination revealed a triangular wound penetrating through the scalp layers, accompanied by extensive bruises indicative of trauma. Notably, air embolism was discovered in the venous and cardiac systems, primarily attributed to injury of the temporal vein near the head wound. This complication was most likely exacerbated by an underlying coagulation disorder stemming from mixed nodular cirrhosis of the liver. The failure of damaged veins to thrombose and inadequate occlusion of venous lumens might have further facilitated air ingress into the venous system, culminating in a fatal outcome. This case underscores the importance of recognizing and promptly managing head injuries, especially in patients with a history of substance abuse and coexisting medical conditions such as liver disease. Improved awareness and education regarding the risks of substance abuse and the significance of seeking timely medical intervention for traumatic injuries are crucial in preventing similar tragic consequences in the future.

## Introduction

Venous air embolism, or pulmonary air embolism, is a rare yet profoundly consequential phenomenon characterized by the intravascular introduction of air, precipitating the formation of gas emboli within the pulmonary vasculature and cardiac chambers [1]. Etiologically, it often arises from traumatic vascular injuries or medical procedures, notably involving the insertion of catheters, minimally invasive surgeries, and various surgical operations, especially in neurosurgery, heart surgery, and vascular procedures. Specific veins, such as the dural sinuses, are prone to allowing air in, especially when pressure differences lead to embolism [2]. It occurs when there is a compromise in vascular integrity, allowing air to enter, and when pressure differences trap the air [3]. Traumatic injuries or medical procedures can cause the former, and changes in intrathoracic pressure and patient positioning often cause the latter. Procedures performed with the patient in an elevated position, especially in neurosurgery, where the surgical field is above the heart, increase the risk of embolism due to gravity aiding air movement into the veins [4-6]. In addition, particular veins, such as the dural sinuses, are more prone to trapping air, adding to the risk in neurosurgery. The varied symptoms of venous air embolism, from subtle neurological issues to severe heart and lung problems, make diagnosis challenging and require a high level of suspicion [7]. For a fatal air embolism to occur, a volume of air ranging from at least 75 cm^3^ to 200-300 cm^3^ must enter the bloodstream [8]. Venous air embolism presents challenges in diagnosis and treatment due to its slow onset and potential for severe consequences. Here, we present a case in which a significant venous air embolism was discovered during a postmortem examination, prompting an investigation into the factors and mechanisms involved.

## Case presentation

**Figure 1:**
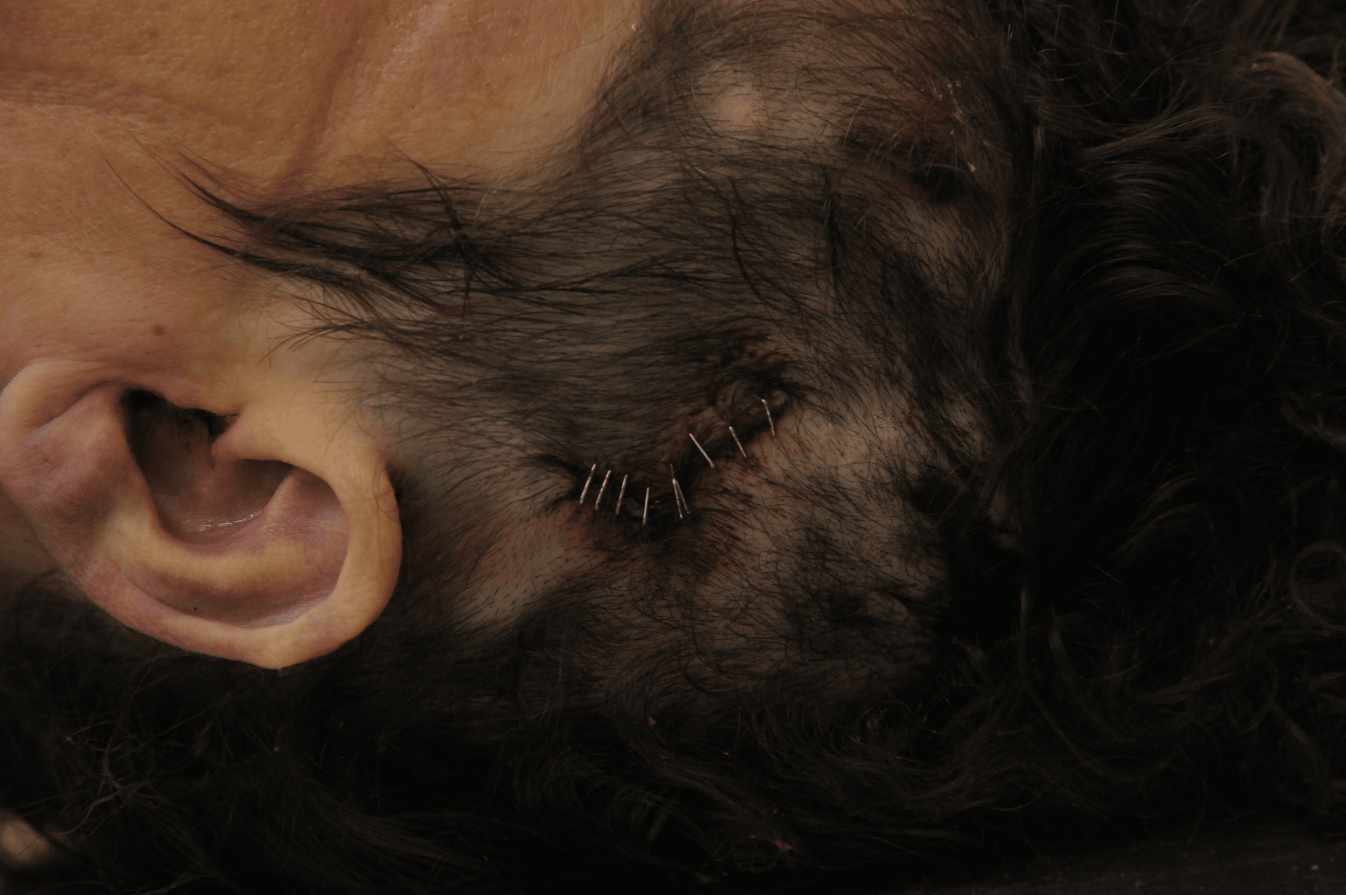
The laceration sutured with surgical clamps.

Emergency services were called in the evening at approximately 10 p.m. for a 34-year-old female patient with a history of substance abuse following a head injury. Amid a domestic dispute with her partner, she threw objects around the apartment while intoxicated. However, she inadvertently slipped and fell, hitting her head on a glass resting on the floor. When the emergency response team arrived, it was noted that the injured woman was experiencing significant bleeding from a wound on her head, and she was initially resistant to medical assistance due to her intoxicated state. Eventually, she agreed to have her head wound treated, but she refused transportation to the hospital for further care. At 2 a.m., another ambulance was requested because the head wound continued to bleed. Upon assessment in the emergency department, a wound was identified on the left parietal region of her head, with blood seepage and a surrounding hematoma. X-ray imaging was not conducted due to the patient's combative and restless behavior. The head wound was managed with staples (Figure 1).

Upon admission to the hospital, the patient's blood pressure was recorded as 91/63 mmHg (heart rate: 93/minute), gradually increasing during her hospitalization. The latest measurement at 5 a.m. showed a blood pressure of 143/63 mmHg (heart rate: 61/minute). Due to vessel occlusion, venous access could not be established, and the patient remained prone in the ward without receiving further treatment until she independently rose around 6 am to use the toilet. Shortly afterward, staff found her lying lifeless on the floor in front of the toilet door, and after 30 minutes of resuscitation, death was pronounced [9,10].

Autopsy findings

**Figure 2:**
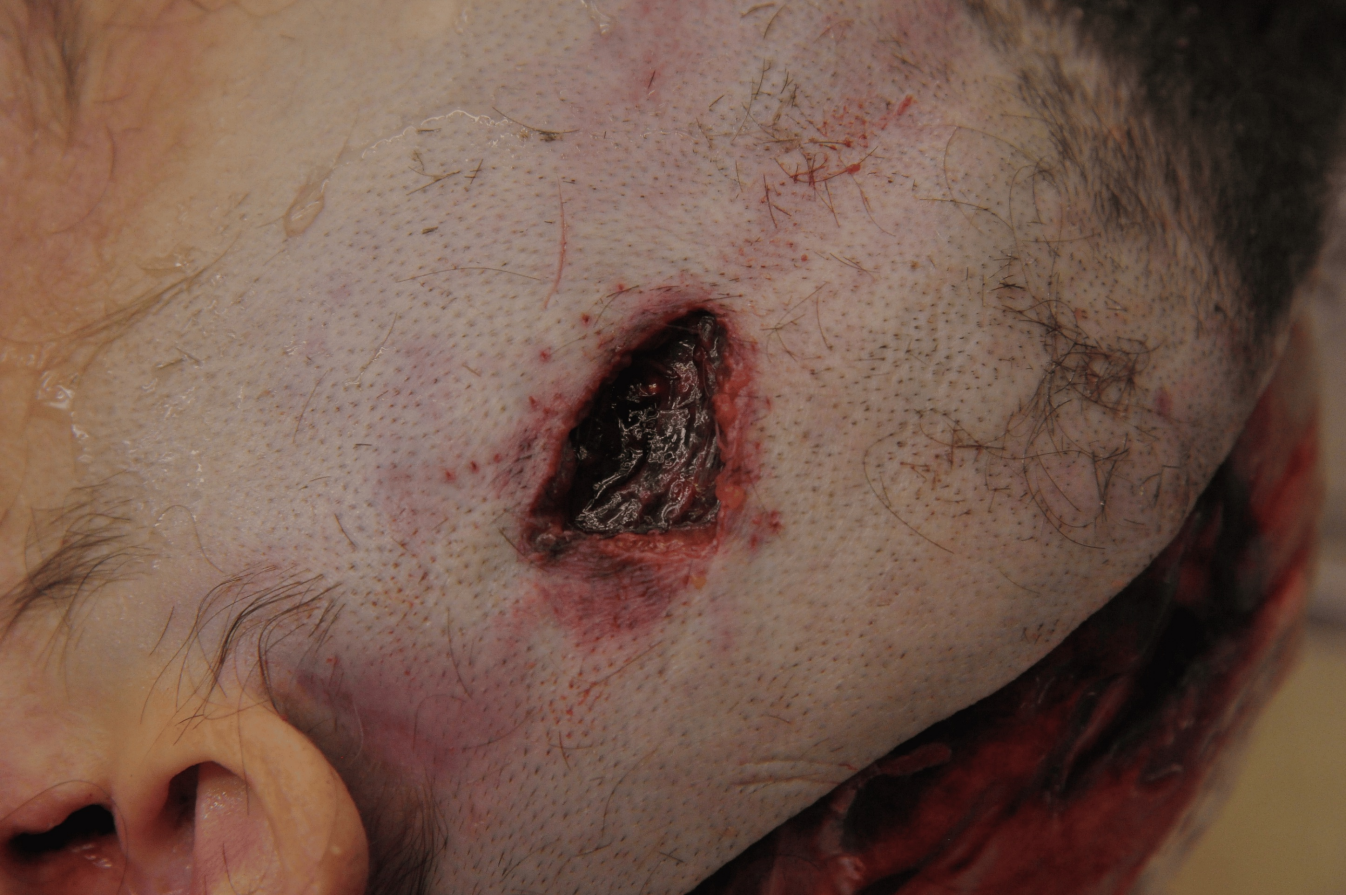
The wound following clamp removal.

**Figure 3:**
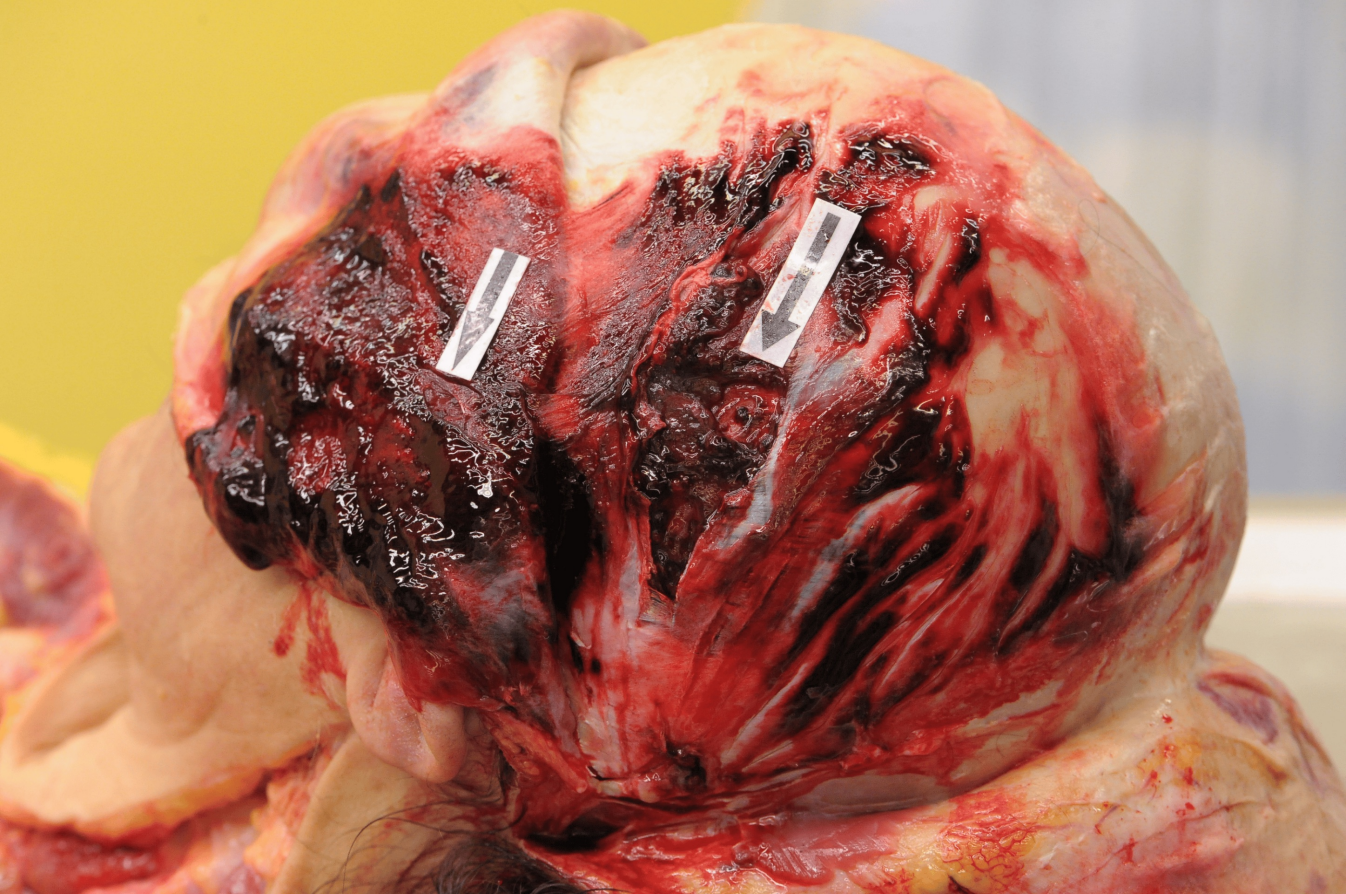
The depth of the injury.

The autopsy was performed 30 hours after death, with an interval of three hours during which the body was not refrigerated. The examination revealed a significant number of bruises and hematomas of various ages distributed across the body; however, the sole open wound was noted on the left parietal region of the head. Upon removal of surgical staples and shaving of surrounding hair, a triangular wound measuring 2.0 × 2.5 × 3.4 cm was identified (Figure 2), penetrating through the total thickness of the scalp (skin, connective tissue, galea aponeurosis, loose areolar connective tissue, a portion of the left superficial temporal muscle, and the pericranium, which covers the outer cortical bone of the calvaria) (Figure 3).

**Figure 4:**
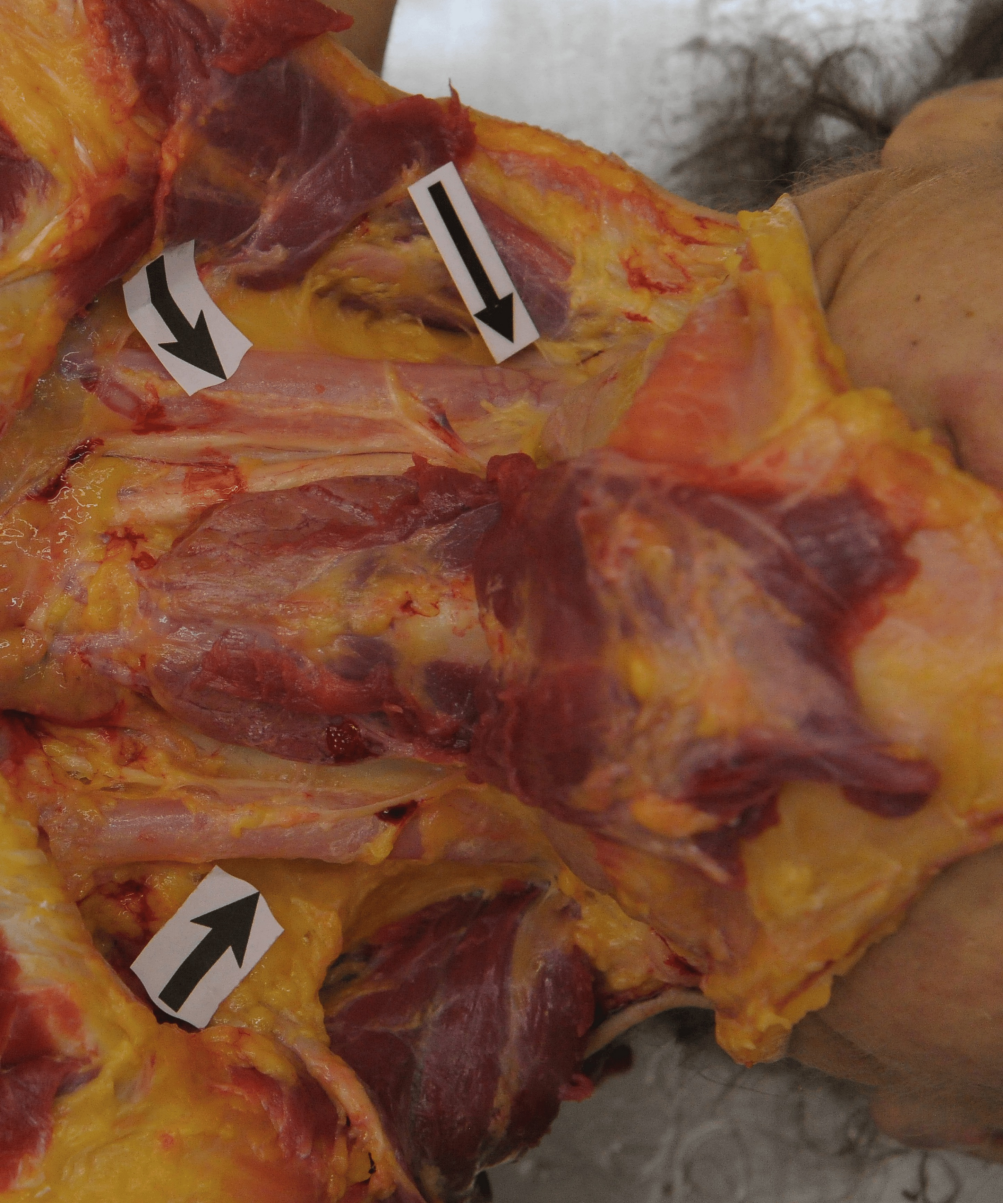
The presence of air in the jugular veins was noticed after the neck dissection, before any bodily cavity was opened.

**Figure 5:**
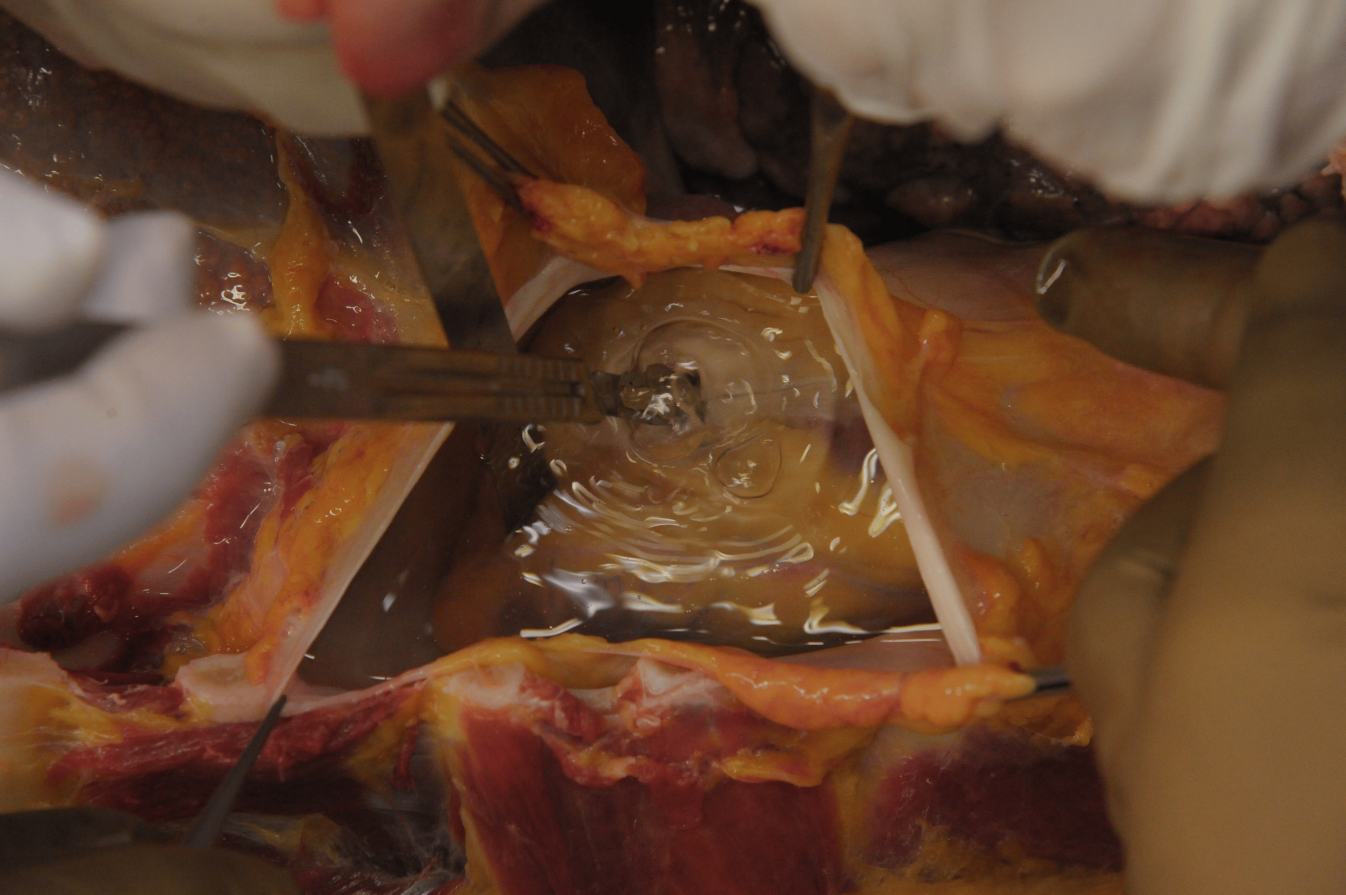
Positive pericardial water test.

Subsequent examination of the soft tissues and skull revealed no evidence of fractures. Following the dissection of the neck muscles, it was noted that the jugular veins on both sides were distended with air (Figure 4). Puncturing the heart in the water-immersed right atrium, which was the first cavity to be opened during the autopsy, revealed the presence of a dense pocket of air bubbles (Figure 5).

**Figure 6:**
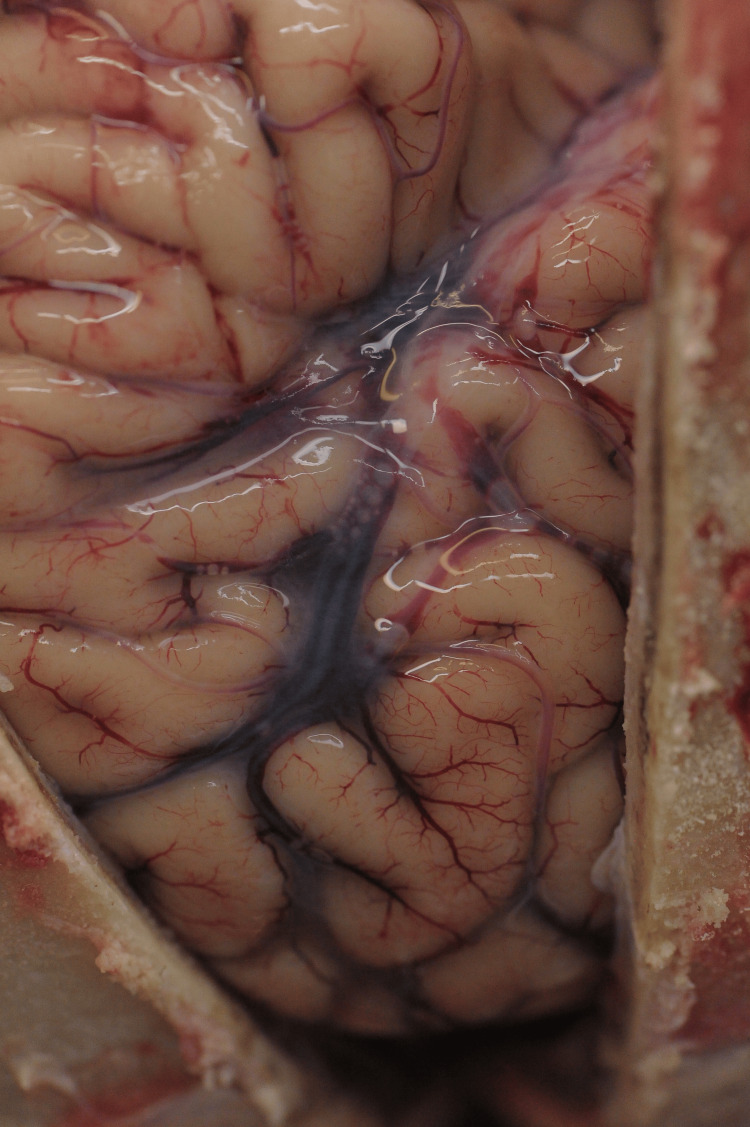
Air embolism affecting cerebral veins.

Upon inspecting the brain, it was noted before brain removal that air bubbles arranged in a bead-like fashion were observed within the venous vessels of the soft meninges between the blood columns in the area of the left and right insulae (Figure 6).

Upon brain removal, clear cerebrospinal fluid was drained out, and an examination of the skull base revealed no fractures. In addition to physical injuries, mixed nodular cirrhosis of the liver and cholecystolithiasis were identified as coexisting conditions. At the time of death, the deceased had 2.97 g/kg of ethyl alcohol in her blood and 4.04 g/kg in her urine, along with a confirmed presence of THC (tetrahydrocannabinol) at an estimated concentration of 17 ng/mL in her blood, detected using HS-GC-FID (headspace gas chromatography with flame ionization detection) and LC-MS/MS (liquid chromatography with tandem mass spectrometry) methods. Based on the autopsy findings, it was concluded that the air embolism stemmed from damage to a vein associated with the head laceration. The delay in the management of the head wound, coupled with a potential coagulopathy associated with liver cirrhosis, may have contributed to the observed complication, with venous air embolism resulting from unclosed veins identified as the ultimate cause of death. Such an outcome, particularly with delayed wound treatment, would be improbable in a healthy individual without a clotting disorder [11].

## Discussion

The failure of the damaged veins in the head to clot, along with the probability that the initial scalp staples did not entirely block the damaged vein openings, allowed air to continue entering the venous system because of negative pressure. This led to air buildup in the right cardiac chambers and pulmonary veins, resulting in death by air embolism.

The case illuminates the intricate interplay between trauma, underlying pathology, and delayed medical intervention, leading to a fatal outcome. Following a head injury during an altercation while intoxicated, the patient initially resisted medical assistance, but she later agreed to the treatment of a significant head wound. Autopsy findings revealed a triangular wound penetrating through the scalp layers, with accompanying bruises suggestive of extensive trauma. Notably, air embolism was discovered in the venous and cardiac systems, primarily attributed to injury of the temporal vein near the head wound. This complication was probably exacerbated by an underlying coagulation disorder stemming from mixed nodular cirrhosis of the liver. The failure of damaged veins to thrombose and the inadequate occlusion of vein lumens further facilitated air ingress into the venous system, culminating in a fatal outcome. This case underscores the imperative of recognizing and promptly managing head injuries, especially in patients with a history of substance abuse and coexisting medical conditions such as liver disease. Although air embolism is an established complication of head injury, possessing a well-differentiated diagnostic sequence and therapy [12], it occasionally remains unrecognized.

The physiological impact of venous air embolism is associated with the air accumulated in the right ventricle. In living cases, clinical observations can range from localized neurological impairments to seizures, unconsciousness, and widespread brain dysfunction. In addition, air embolism may induce significant cardiovascular and respiratory issues. A significant embolism could result in abrupt cardiovascular failure due to complete blockage of output from the right ventricle, reduced input from pulmonary veins, sudden failure of the right heart, diminished preload in the left ventricle, or decreased cardiac output [13,14].

If the condition had been identified, prompt medical intervention could have improved the patient's chances of survival. Treatment measures might have involved placing her in the correct position, administering oxygen, aspirating air from the right atrium, and possibly hyperbaric therapy [15].

Improved awareness and education regarding the risks of substance abuse and the significance of seeking timely medical intervention for traumatic injuries are pivotal in averting similar tragic consequences in the future. In this case, discovering a significant venous air embolism during a postmortem examination emphasizes the need for careful analysis of predisposing factors and events. The interaction of patient health issues, complex procedures, and anatomical vulnerabilities highlights the importance of thorough risk assessment and strict adherence to preventative measures.

## Conclusions

This case report illustrates the critical need for vigilance and prompt medical intervention in patients with head injuries, particularly those with a history of substance abuse and underlying health conditions such as liver cirrhosis. The unexpected finding of venous air embolism in the context of a traumatic head injury underscores the complex interplay between trauma, delayed treatment, and coagulopathy. In this case, the combination of an untreated head wound and compromised vascular integrity resulted in a fatal outcome exacerbated by the patient's preexisting mixed nodular cirrhosis. This report highlights the importance of timely assessment and management of head injuries, as well as the necessity for heightened awareness of the risks associated with substance use. Educating healthcare providers and the public about these risks is essential for preventing similar tragedies in the future. Overall, this case serves as a poignant reminder of the potentially catastrophic consequences of neglecting the signs and symptoms associated with trauma, particularly in vulnerable populations.

## References

[1] Adams V, Guidi C (2001). Venous air embolism in homicidal blunt impact head trauma. Case reports. Am J Forensic Med Pathol.

[2] Brune JE, Kaech DL, Wyler D, Jeker R (2018). Delayed lethal pulmonary air embolism after a gunshot head injury. BMJ Case Rep.

[3] McCarthy CJ, Behravesh S, Naidu SG, Oklu R (2016). Air embolism: practical tips for prevention and treatment. J Clin Med.

[4] Rozet I, Vavilala MS (2007). Risks and benefits of patient positioning during neurosurgical care. Anesthesiol Clin.

[5] Marsh PL, Moore EE, Moore HB (2023). Iatrogenic air embolism: pathoanatomy, thromboinflammation, endotheliopathy, and therapies. Front Immunol.

[6] Bahloul M, Dlela M, Bouchaala K, Kallel H, Ben Hamida C, Chelly H, Bouaziz M (2020). Post-traumatic pulmonary embolism: incidence, physiopathology, risk factors of early occurrence, and impact outcome. A narrative review. Am J Cardiovasc Dis.

[7] Bokhari R, You E, Bakhaidar M (2020). Dural venous sinus thrombosis in patients presenting with blunt traumatic brain injuries and skull fractures: a systematic review and meta-analysis. World Neurosurg.

[8] Toung TJ, Rossberg MI, Hutchins GM (2001). Volume of air in a lethal venous air embolism. Anesthesiology.

[9] Campkin TV, Perks JS (1973). Venous air embolism. Lancet.

[10] Maule S, Milazzo V, Maule MM, Di Stefano C, Milan A, Veglio F (2012). Mortality and prognosis in patients with neurogenic orthostatic hypotension. Funct Neurol.

[11] Islam R, Kundu S, Jha SB (2022). Cirrhosis and coagulopathy: mechanisms of hemostasis changes in liver failure and their management. Cureus.

[12] Brown AE, Rabinstein AA, Braksick SA (2023). Clinical characteristics, imaging findings, and outcomes of cerebral air embolism. Neurocrit Care.

[13] Roquero LP, Camelo-Piragua S, Schmidt C (2016). Cerebral air embolism: a clinical, radiologic and histopathologic correlation. Am J Forensic Med Pathol.

[14] Petekkaya S, Celbiş O, Oner BS, Turan Ö, Yener Z (2019). A rare case of fatal venous and cerebral air embolism. Ulus Travma Acil Cerrahi Derg.

[15] Shaikh N, Ummunisa F (2009). Acute management of vascular air embolism. J Emerg Trauma Shock.

